# Effects of probiotic bacteria, isoflavones and simvastatin on lipid profile and atherosclerosis in cholesterol-fed rabbits: a randomized double-blind study

**DOI:** 10.1186/1476-511X-8-1

**Published:** 2009-01-07

**Authors:** Daniela CU Cavallini, Raquel Bedani, Laura Q Bomdespacho, Regina C Vendramini, Elizeu A Rossi

**Affiliations:** 1 Department of Food & Nutrition, Faculty of Pharmaceutical Sciences, Sao Paulo State University, Araraquara, SP, Brazil; 2 Department of Clinical Analysis, Faculty of Pharmaceutical Sciences, Sao Paulo State University, Araraquara, SP, Brazil

## Abstract

**Background:**

Much attention has been drawn to different alternative strategies for cardiovascular disease prevention. Objective: The aim of the present study was to observe and compare the effects of *Enterococcus faecium *CRL183 (probiotic microorganism), an isoflavones mixture and simvastatin (drug used to treat hypercholesterolemia) on lipid parameters and atherosclerosis development in rabbits with induced hypercholesterolemia.

**Methods:**

The animals were randomly allocated to 5 experimental groups (n = 6) for 60 days: control (C) that did not consume cholesterol, hypercholesterolemic (H) that consumed an atherogenic diet (1.0% cholesterol wt/wt), hypercholesterolemic plus *E. faecium *(HE), hypercholesterolemic plus isoflavone (HI) and hypercholesterolemic plus simvastatin (HS). Total and HDL-cholesterol and triglycerides were determined by enzymatic methods; non-HDL-C was calculated by subtracting HDL-C from total cholesterol; and atherosclerosis was presented as the percentage of lesion area, relative to the total area from the aorta segment analyzed.

**Results:**

Simvastatin significantly reduced the tot cholesterol (16%) and non-HDL-C level (17%) and increased the HDL-C (98%), compared to group H. *E. faecium *raised the HDL-C level by 43.3% (P < 0.05). Isoflavone decreased the total cholesterol and non-HDL-C concentrations (9%), but this effect was not statistically significant. At the end of the treatments, groups HE and HS had significantly lower levels of triglycerides in relation to H and HI. The atherosclerotic lesion area in the aortic arch was not different between groups. The extent of atherosclerosis in the thoracic and abdominal aorta was reduced in the groups HI and HS by 22.7% and 26.7% respectively, but this effect was not significant (P > 0.05).

**Conclusion:**

The results indicated that probiotic microorganism *E. faecium *CRL 183 could be used to improve the lipid profile as an alternative or an adjuvant for drug therapy. The effectiveness of simvastatin in the management of blood lipid was confirmed. There were no effects of soy isoflavones, *E. faecium *and simvastatin on atherosclerosis development.

## Background

Cardiovascular diseases (CVD) are the most common cause of death around the world. Elevated blood lipid levels are a major determinant of coronary heart disease (CHD) and others atherosclerotics diseases. Therefore, the control of hyperlipidemia may lower CVD risk [1,2].

3-Hidroxy 3-metylglutaryl coenzyme A (HMG-CoA) redutase inhibitors, know also as statins, are considered the most effective drugs in the management of hypercholesterolemia and prevention of atherosclerosis-related disorders [3,4]. Statins are selective inhibitors of HMG CoA redutase, the rate-limiting enzyme of cholesterol biosynthesis, reducing low-density lipoprotein (LDL), very low-density lipoprotein (VLDL) and triglyceride levels [5]. Besides lipid-lowering, statins have additional effects on atherosclerosis vascular disease, that includes anti-inflammatory [6], anti-thrombotic properties [7] and improve of endothelial function [8].

Currently the high cost of medicines is a limitation for pharmacological therapy adhesion. Therefore, alternative strategies for CVD prevention, such as dietary therapy, have received considerable attention of scientific community.

Several studies in animals and humans have shown that soy and soy isoflavones may protect against CVD through improves on serum lipid profiles [9,10] and vascular reactivity [11], increases of LDL oxidation resistance [12] and inhibition thrombus formation [11].

Among the beneficial effects attributed to probiotics and probiotic-containing food products, the reduction of blood cholesterol is of particular interest. The primary probiotic bacteria associated with cholesterol lowering have been lactobacilli and bifidobacteria, although other lactic acid bacteria, such as enterococci, are able to produce this effect [9,13,14].

We demonstrated previously that *E. faecium *CRL183 reduces cholesterol by 53.85% in an *in vitro *model [15]. We also showed that a soy product fermented with *E. faecium *CRL 183 and *Lactobacillus helveticus *ssp *jugurti *416 exhibited a significant hypocholesterolemic effect in animal tests and clinical trials [16-18]. However the hypocholesterolemic effect of *E. faecium *CRL 183 strain has not been studied *in vivo *model.

Hence, the aim of the present study was to verify and compare the effects of *E. faecium *CRL183, isoflavones and simvastatin on lipid parameters and atherosclerosis development in rabbits with induced hypercholesterolemia.

## Methods

### Material

*Enterococcus faecium *CRL 183 was obtained from Reference Center for Lactobacilli – CERELA (San Miguel de Tucumán, Argentina), isoflavone mixture (Isoflavin^®^: 4.7% genistin, 11.3% genistein, 5.5% daidzin, 17.8% daidzein, 2.0% glycitin and 1.0% glycitein) were purchased from Galena, (Campinas, SP, Brazil) and simvastatin were purchased from Galena (Campinas, SP, Brazil).

### Animals and Diets

The animal experimental protocol received prior approval from the Research Ethics Committee of the School of Pharmaceutical Sciences (n° 03/2007 – UNESP at Araraquara, SP, Brazil). New Zealand white male rabbits (n = 30), 8–9 weeks old, weighing 2.5–3.0 Kg, were obtained from Central Biotery of Sao Paulo State University, Botucatu, SP, Brazil. They were housed individually in temperature-controlled rooms (22°C) with a light-dark cycle of 12:12 h. Rabbits were fed a chow diet (Purina, SP, Brazil) for 1 week to acclimate the animals and then randomly allocated to 5 experimental groups (n = 6): control (C), hypercholesterolemic (H), hypercholesterolemic plus *E. faecium *(HE), hypercholesterolemic plus isoflavone (HI) and hypercholesterolemic plus simvastatin (HS). The control group (C) was fed only with commercial rabbit diet (Nutri Coelhos Especial Purina), with the following nutritional make-up (per 100 g): 23 g protein, 4 g fats, 49 g carbohydrates, 5 g fiber and 10 g minerals. The other groups (H, HE, HI and HS) were fed on the same rabbit diet, to which cholesterol (Sigma C 8503) had been added to induce hypercholesterolemia. The level of cholesterol added to the diet was adjusted during the protocol (1.0% to 0.7% after 30 days) to maintain the animal health. To prepare the supplemented diet, ether-diluted cholesterol (ethyl ether stabilized with BHT, Carlo Elba, Italy), was pulverized as a fine mist on to the chow, under a hood, where it remained for 12 h to allow the solvents to evaporate completely. The chow was packed in black plastic bags and stored -10°C no more than 2 weeks before use. The rabbits received restrict amounts (125 g/d) of each diet, because the extent of atherosclerosis depends on cholesterol intake [19]. The animals had free access to water during the experimental period. Groups HE, HI and HS were given, by gavage once a day, *E. faecium *suspension (10^8 ^CFU), isoflavone (2.1 mg/kg of body weight) and simvastatin (3.0 mg/kg of body weight), respectively. To prepare the pure culture of probiotic microorganism, *E. faecium *CRL 183 was reinoculated into Tryptic Soy Broth (Acumedia) and incubated at 37°C for 16 hours. The cells were centrifuged at 3000 rpm for 5 minutes and the supernatant discarded. The cells were resuspended in sterile peptone water and the suspension was stored under refrigeration until administered to the animals. Isoflavone mixture and simvastatin were diluted in sterile water immediately before use.

All animals were fed experimental diets for 60 days and were weighed 3 times during the study (0, 30 and 60 days). Food intake was measured daily. Blood were drawn from the marginal ear veins, after a 14–16-hour fast, at 0, 30 and 60 day of treatment. Samples were centrifuged (3500 × g for 10 min at 4°C) to separate serum that was stored (-70°C) until analysis. At the end of 60 d, the rabbits were heparinized (Roche, SP, Brazil) and killed by an overdose of sodium phenobarbital (Cristália, SP, Brazil). Immediately after the sacrifice the liver, kidneys and heart were removed and weighed. The role aorta was removed from its origin (valve aortic) down to the bifurcation of the internal iliac arteries to analysis of atherosclerotic lesions.

### Determination of the Coefficient of Alimentary Efficacy (CAE)

The CEA was defined as the ratio of the difference in body weight between days 60 and 0 and the amount of food consumed throughout the experimental period.

### Analysis of Serum lipids

The serum levels of TC, HDL-C and triglycerides were assayed in each rabbit, with the aid of specific enzyme kits. Total cholesterol was measured by the cholesterol fast color method [20]. HDL cholesterol was estimated by first selectively precipitating lipoproteins [21] and then applying the TC method to the supernatant. Triglycerides were measured by the triglyceride fast color method [22]. Non-HDL cholesterol was calculated by subtracting HDL-C from TC and represented the LDL+IDL+VLDL cholesterol fractions [23,24].

### Analysis of Atherosclerotic Lesions

The aorta was divided into two segments comprising: 1) arch aortic; 2) thoracic aorta and abdominal aorta. The material was fixed, overnight at room temperature, in 10% buffered formalin solution and stained with Sudan IV to visualize areas of atherosclerotic plaque [25]. The stained aorta was photographed with a digital camera (Sony) and the sudanophilic lesions were identified and quantified. The surface area of the atherosclerotic lesions was measured with an image analyzer system (Imagelab – USP – Brazil) and expressed as a percentage of the total surface area covered by lesion, on the arch and thoracic aorta.

### Statistical Analysis

Results are expressed as mean ± standard error of the mean. The data were tested by analysis of variance (ANOVA) and the means were compared across groups by Tukey test, significance being declared when P ≤ 0.05. The relationship between the percent lesion area and the blood lipids was determined by linear correlation analysis. All analyses were carried out with the BIOSTAT statistical package.

## Results

### Food intake, weight gain and organ weight

Daily food intake was higher in the rabbits of C and H groups. On the other hand, animals that received isoflavone and simvastatin showed the lowest food intake, during the experiment.

The weight gains of hypercholesterolemic rabbits (group H) tended to be lower those in the other groups and these animals exhibited the lower CAE (p < 0.05).

Dietary isoflavones, probiotic bacteria and simvastatin significantly reduced weights of liver and kidneys compared to group H. Additionally, the rabbits treated with isoflavones had lower heart weight (Table 1).

**Table 1:** Effects of *E. faecium*, isoflavones and simvastatin on food intake, weight gain and organ weight in the rabbits.

G*	**Food Intake** **(g)**	**Weight gain** **(g)**	CAE**	**Liver** **(g)**	**Heart** **(g)**	**Kidney** **(g)**
**C**	125.00 ± 0.00^a^	903.05 ± 124.86^a^	7.23 ± 0.61^a^	83.17 ± 3.92^c^	5.78 ± 0.20^b^	6.59 ± 0.52^b^
**H**	124.93 ± 0.50^a^	713.40 ± 39.75^b^	5.71 ± 0.31^b^	165.90 ± 3.41^a^	7.06 ± 0.56^a^	10.02 ± 0.72^a^
**HE**	118.17 ± 9.92^b^	810.57 ± 95.22^ab^	6.85 ± 0.28^a^	110.12 ± 7.53^b^	6.24 ± 0.39^ab^	7.21 ± 0.46^b^
**HI**	110.14 ± 17.52^c^	851.88 ± 67.04^ab^	7.76 ± 0.58^a^	98.22 ± 6.07^bc^	5.73 ± 0.22^b^	7.36 ± 0.74^b^
**HS**	114.94 ± 13.11^bc^	796.48 ± 79.76^ab^	6.93 ± 0.44^a^	114.73 ± 10.43^b^	6.17 ± 0.47^ab^	7.59 ± 0.26^b^

Values represent mean ± SEM (n = 6).

Statistical comparison of groups: means with identical letters in the same column do not differ significantly (P ≤ 0.05).

*G = Groups: C = control; H = hypercholesterolemic; HE = hypercholesterolemic plus *E. faecium*; HI = hypercholesterolemic plus isoflavone; HS = hypercholesterolemic plus simvastatin.

** Coefficient of Alimentary Efficacy.

### Lipid Profiles

Table 2 summarizes the effects of treatments on serum lipids. After 30 days of the study, H group exhibited the highest concentration of total cholesterol (TC), not differ significantly (p < 0.05) of the group HE. The simvastatin and the isoflavones caused the greatest reductions in TC (34.0% and 19.5% respectively), compared to group H. At the end of the experiment – 30 days after the reduction of the concentration of cholesterol added to the diet (from 1% to 0.7%) – the groups H, HE and HI showed the highest levels of TC without differ among themselves. Simvastatin led to a reduction of 16% for TC compared to the group that consumed only the feed with the cholesterol addition (H).

**Table 2:** Serum lipids among the groups.

**Serum lipids**	**Time**	**C**	**H**	**HE**	**HI**	**HS**
TC (mg/dl)	T0	49.0 ± 3.7^a^	42.0 ± 2.8^a^	46.3 ± 2.7^a^	46.0 ± 1.9^a^	49.0 ± 5.6^a^
	T30	63.3 ± 4.3^d^	3650.0 ± 138.0^a^	3413.5 ± 192.7^a^	2938.5 ± 154.5^b^	2409.3 ± 154.1^c^
	T60	53.3 ± 6.2^c^	2556.5 ± 120.3^a^	2605.0 ± 166.2^a^	2327.0 ± 130.9^ab^	2146.0 ± 172.7^b^
						
HDL-C (mg/dl)	T0	31.5 ± 2.3^a^	28.5 ± 2.5 ^a^	30.3 ± .5 ^a^	29.8 ± 1.9^a^	32.5 ± 4.8^a^
	T30	38.3 ± 2.1^a^	25.5 ± 0.9^c^	34.3 ± 2.7^ab^	30.8 ± 1.8^bc^	40.0 ± 3.1^a^
	T60	26.8 ± 2.8^b^	16.8 ± 2.8^d^	24.0 ± 1.9^bc^	20.5 ± 2.1^cd^	33.3 ± 3.3^a^
						
n-HDL-C (mg/dl)	T0	17.5 ± 2.6^a^	13.5 ± 2.9^a^	16.0 ± 2.0^a^	16.3 ± 1.92^a^	16.5 ± 1.1^a^
	T30	25.0 ± 2.6^d^	3624.5 ± 138.0^a^	3379.3 ± 193.1^a^	2907.8 ± 154.5^b^	2369.3 ± 156.8^c^
	T60	26.5 ± 4.4^c^	2539.8 ± 120.2^a^	2581.5 ± 166.2^a^	2307.0 ± 71.3^ab^	2112.8 ± 183.1^b^
						
Triglycerides (mg/dl)	T0	90.0 ± 5.3^a^	66.0 ± 7.3^b^	99.5 ± 9.5^a^	99.3 ± 9.2^a^	94.0 ± 4.2^a^
	T30	57.5 ± 2.9^d^	260.3 ± 9.4^a^	122.8 ± 20.4^c^	203.3 ± 3.6^b^	110.0 ± 4.7^c^
	T60	53.0 ± 4.5^c^	245.5 ± 25.9^a^	115.8 ± 6.4^b^	215.0 ± 7.1^a^	104.0 ± 17.2^b^

Values (in mg/dL) represent mean ± SEM (n = 6). Statistical comparison of groups: means with identical minuscules letters in the same line do not differ significantly (P ≤ 0.05). C = control; H = hypercholesterolemic; HE = hypercholesterolemic plus *E. faecium*; HI = hypercholesterolemic plus isoflavone; HS = hypercholesterolemic plus simvastatin.

With 30 days of trial, the concentration of HDL-C was lower (P < 0.05) in animals of the groups H and HI. On the other hand, animals in groups HS and HE showed levels of HDL-C significantly higher (56.9% and 34.3%, respectively) to the group H and similar to the control (C). At the end of the protocol, the simvastatin (HS) was able to raise the HDL-C by 98.5% and 24.30% compared to groups H and C, respectively, and this effect was significantly (P < 0.05) higher than found in other groups. The rabbits that received the suspension of *E. faecium *showed an increase of 43.3% in this cholesterol fraction in relation of the group H, without differ from the control group (C). The animals treated with isoflavone exhibited HDL-C concentration similar to the H and HE groups (p ≤ 0.05).

The fraction nHDL-C exhibited a similar behavior to that observed for the CT.

The animals of group H presented basal triglycerides level significantly lower to the other groups (P < 0.05). At the end of the protocol groups HE and HS had significantly lower levels of triglycerides (P < 0.05) compared to groups H and HS.

### Extent of Atherosclerosis

**Figure 1:**
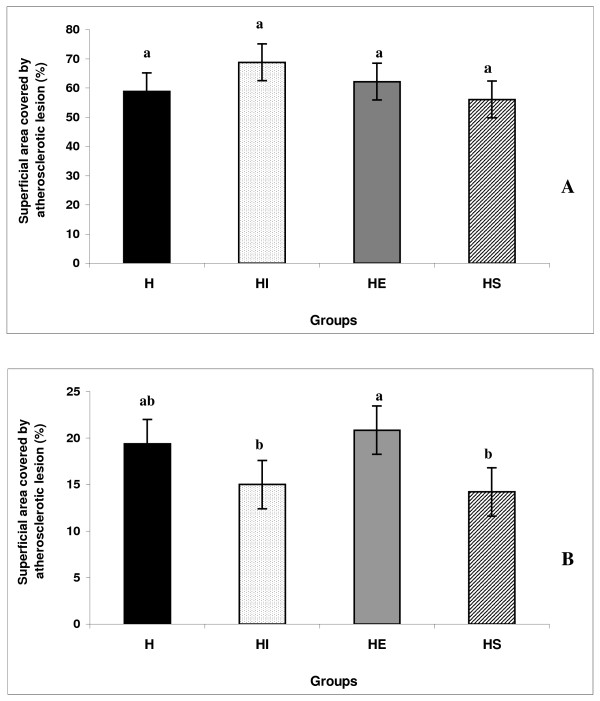
**Percent of aortic area – A = arch segment; B = thoracic segment – covered by lesion**. The bar graphs represent the average (n = 6) for each group with standard errors. H = hypercholesterolemic; HE = hypercholesterolemic plus *E. faecium*; HI = hypercholesterolemic plus isoflavone; HS = hypercholesterolemic plus simvastatin.

None animals on regular diet developed evidence of atherosclerosis. In contrast, all rabbits on cholesterol-enriched diet developed atherosclerosis (Figure 1). The distribution of atherosclerotic lesion over entire aorta was similar in all groups of hypercholesterolemic animals, with the aortic arch contributed between 68% and 83%. The lesion area of the aortic arch did not differ between groups. The extent of atherosclerosis in the thoracic and abdominal aorta was reduced in the HI and HS groups by 22.7% and 26.7%, respectively compared to the group H (no significant P < 0.05). Thoracic and abdominal lesions area was positively correlated with TC and non-HDL-C concentrations (Table 3).

**Table 3:** Correlations of serum lipids and atherosclerotic lesions

**Serum Lipids**	**Pearson Correlation Coefficients (r)**	
	
	**Aortic Arch**	**Thoracic Aorta**
Total Cholesterol	-0.08	0.99
HDL-C	-0.49	-0.52
nHDL-C	0.24	0.92
Triglycerides	0.32	0.19

## Discussion

The cholesterol added to the chow diet induced hypercholesterolemia in groups H relative to control animals and none of treatments was able to reducing the serum lipids to basal levels.

After 30 days, the soy isoflavones reduced by 19.5% the TC and n-HDL-C. However, this effect was not maintained until the end of the protocol. Several studies have evaluated the effect of isoflavones, associated or not with the soy protein, in lipid levels and the results are not uniform. Song et al. (2006) [23] observed in hamsters that soy protein with or without isoflavones and daidzein produced similar plasma cholesterol-lowering effects. Lee et al. (2007) [26] demonstrated that glycitein lessened plasma cholesterol in female hamsters compared with a casein control diet, an effect not observed with daidzein and genistein at the same doses.

Previous study showed that the *E. faecium *CRL 183 was capable of reducing total cholesterol by 54% in an *in vitro *model [15]. However, the results of this work did not confirm the effect of pure culture of *E. faecium *CRL 183 in animal model. Rossi *et al*. (1999) [16] developed a soy yogurt, fermented by *Enterococcus faecium *CRL 183 (probiotic microorganism) and *Lactobacillus helveticus *ssp *jugurti *416. This product exhibited a significant hypocholesterolemic effect in clinical trials and animal tests [17,18]. It is possible that the components of soy yogurt and the bioactive compounds produced during the fermentative process have been involved in these lowering-cholesterol effects.

Statins competitively inhibit HMG-CoA reductase, the rate-limiting enzyme of the mevalonate pathway, thereby decreasing intra-cellular cholesterol synthesis. The resulting decrease in hepatic intra-cellular cholesterol concentration results in compensatory increase in the expression of hepatic LDL receptors, which clear LDL from the circulation [5]. In this study the rabbits treated with simvastatin showed a reduction of 16% in cholesterol total and non-HDL-C levels compared to hypercholesterolemic rabbits (group H), though serum cholesterol levels remain much higher than in normolipidemic animals. Our results are in agreement with those observed by others researchers. Al-Zuhair et al. (1997) [27] showed that simvastatin (1.86 mg/Kg, twice daily) produced significant reductions by 17%, 31.5% and 21% in LDL-C, triglycerides and total cholesterol levels in cholesterol-fed rabbits (0.5% wt/wt diet). Shiomi et al. (2004) [28] demonstrated that simvastatin (15 mg/Kg/52 days) reduced the total cholesterol by about 20%in rabbits.

Probiotic bacteria (*E. faecium *CRL 183) was able to prevent the reduction of HDL-C compared to control group and to raise this lipoprotein compared to group H (43.3%). We demonstrated previously that a soy product fermented with *E. faecium *CRL 183 increased by 18% and 10% the concentration of HDL-C of health rabbits and adults men, respectively [17,18].

Simvastatin promoted an increase of 98.5% in HDL-C compared to group H. These results are higher than those recorded in literature, where the administration of statins results in a modest increase in HDL-C (5% to 10%) [3,4].

In this study, simvastatin and probiotic microorganism prevented the elevation of triglycerides during the protocol. Previous studies showed that soy yogurt fermented with *E. faecium *CRL 183 did not alter the triglycerides level in animals and humans [17,18]. On the other hand, the HMG-CoA reductase inhibitors have been considered effective at lowering triglyceride – or more specifically, VLDL triglyceride – levels [29,30].

The correlation between blood cholesterol and the extent of atherosclerosis has been well established in human and animal model [31,32]. We observed a positive correlation between TC, n-HDL-C and extent of atherosclerosis lesion in the thoracic-abdominal aorta.

In the present study soy isoflavone and simvastatin tended to reduce the atherosclerostic lesion in cholesterol-fed rabbits, but this effect was not significant (P < 0.05). This data not confirm the earlier findings by others that isoflavones and simvastatin reduce atherosclerosis development in animals. Yamakoshi et al. (2000) [19] reported that the extent of atherosclerosis in the aortic arch of cholesterol-fed rabbits was significantly reduced in the isoflavone groups (26.3% – 36.9%) Fukuo et al (1991) [33] observed that the extent of atherosclerosis lesions on thoracic-abdominal aorta was lower in the simvastatin (10 mg/Kg) treated animals than in the controls, but the difference was significant only in the young rabbits (3 months). Differences on experimental design could be responsible to the observed results in this study.

The potential effects of isoflavones on CDV protection include modulation of pro-inflammatory cytokines; cell adhesion proteins and NO formation; protection of LDL against oxidation; inhibition of platelet aggregation and improvement in vascular reactivity [9-11]. The discovery of statins led to important improvements in prevention of CDV diseases. Recent data suggest that apart their lipid-lowering effects, statins have a range of anti-inflammatory and anti-thrombotic properties and seem to improve endothelial function [5]. Additional studies are necessary to confirm the effect of isoflavone and simvastatin on atherosclerosis development.

## Conclusion

In conclusion, the results indicated that the probiotic microorganism *E. faecium *CRL 183 could be used to improve lipid profile as an alternative or an adjuvant for drug therapy. Simvastatin confirmed its effectiveness in the management of blood lipid. There were no significant effects of soy isoflavones, *E. faecium *and simvastatin on atherosclerosis development.

## Abbreviations

CVD: Cardiovascular Disease; CHD: Coronary Heart Disease; C: Control Group; H: Hypercholesterolemic Group; HE: Hypercholesterolemic plus *E. faecium *Group; HI: Hypercholesterolemic plus Isoflavone Group; HS: Hypercholesterolemic plus Simvastatin Group; CAE: Coefficient of Alimentary Efficacy; TC: Total Cholesterol; HDL-C: High Density Lipoprotein Cholesterol; LDL-C: Low Density lipoprotein Cholesterol; VLDL: Very Low Density lipoprotein Cholesterol; n-DHL-C: Non High Density Lipoprotein Cholesterol; HMG-CoA reductase: 3-Hidroxy 3-metylglutaryl coenzyme A reductase.

## Competing interests

The authors declare that they have no competing interests.

## Authors' contributions

DCUC has been involved in design, data collection, drafting the manuscript and revising it critically for important intellectual content. RB, LQB, RCV have been involved in data collection and drafting the manuscript. EAR has been involved in design, drafting the manuscript and revising it critically for important intellectual content.
